# Diarrhea Predominant-Irritable Bowel Syndrome (IBS-D): Effects of Different Nutritional Patterns on Intestinal Dysbiosis and Symptoms

**DOI:** 10.3390/nu13051506

**Published:** 2021-04-29

**Authors:** Annamaria Altomare, Claudia Di Rosa, Elena Imperia, Sara Emerenziani, Michele Cicala, Michele Pier Luca Guarino

**Affiliations:** 1 Gastroenterology Unit, Campus Bio-Medico University of Rome, Via Álvaro del Portillo 21, 00128 Rome, Italy; a.altomare@unicampus.it (A.A.); s.emerenziani@unicampus.it (S.E.); m.cicala@unicampus.it (M.C.); m.guarino@unicampus.it (M.P.L.G.); 2 Unit of Food Science and Human Nutrition, Campus Bio-Medico University of Rome, Via Álvaro del Portillo 21, 00128 Rome, Italy; elenaimperia.96@gmail.com

**Keywords:** Irritable Bowel Syndrome, IBS, IBS-D, diarrhea predominant IBS, microbiota and IBS, diet and IBS, low-FODMAP and IBS, low-FODMAP and microbiota, gluten-free diet and IBS, gluten-free diet and microbiota

## Abstract

Irritable Bowel Syndrome (IBS) is a chronic functional gastrointestinal disorder characterized by abdominal pain associated with defecation or a change in bowel habits. Gut microbiota, which acts as a real organ with well-defined functions, is in a mutualistic relationship with the host, harvesting additional energy and nutrients from the diet and protecting the host from pathogens; specific alterations in its composition seem to play a crucial role in IBS pathophysiology. It is well known that diet can significantly modulate the intestinal microbiota profile but it is less known how different nutritional approach effective in IBS patients, such as the low-FODMAP diet, could be responsible of intestinal microbiota changes, thus influencing the presence of gastrointestinal (GI) symptoms. The aim of this review was to explore the effects of different nutritional protocols (e.g., traditional nutritional advice, low-FODMAP diet, gluten-free diet, etc.) on IBS-D symptoms and on intestinal microbiota variations in both IBS-D patients and healthy subjects. To date, an ideal nutritional protocol does not exist for IBS-D patients but it seems crucial to consider the effect of the different nutritional approaches on the intestinal microbiota composition to better define an efficient strategy to manage this functional disorder.

## 1. Introduction

Irritable Bowel Syndrome (IBS) is a chronic functional gastrointestinal (GI) disorder characterized by abdominal pain associated with defecation or a change in bowel habits [1]. It affects between approximately 5% and 15% of the Western Population and it is predominant in women younger than 50 years old (11.2% prevalence) but it can occur at any age [2,3]. IBS is characterized by several GI symptoms like abdominal pain, bloating, urgency, a sensation of incomplete evacuation and altered bowel habits without any organic and biochemical abnormalities [2,3]. IBS also has a significant negative impact on quality of life (QoL) and it can affect both work and daily activities of patients [2,4,5]. The diagnosis is symptoms-based following the exclusion of other GI disorders detected by radiologic or endoscopic tests [2,3]. The pathophysiology of IBS has not been clearly understood yet, but several factors have been involved such as alterations in gastrointestinal motility, visceral hypersensitivity, small intestinal bacterial overgrowth (SIBO), environmental factors, first of all dietary habits, and intestinal microbiota alterations (dysbiosis) which is still unclear if it is a cause or a consequence in the pathogenesis of this syndrome [2,3].

Among the genetic variations, recent studies have evaluated the hypomorphic sucrase—isomaltase (SI) gene variation as a mechanism involved in IBS, especially IBS-D symptoms [6]. SI deficiency is a condition in which sucrase—isomaltase, an enzyme produced by the brush border of the small intestine to metabolize sucrose, is deficient. This change in small intestine epithelial cells results in a colonic accumulation of undigested disaccharides derived from starch and sucrose and then fermented producing gas that contributes to the generation of abdominal pain, bloating and osmotic diarrhea [6,7]. According to Kim et al. this deficiency should be considered in the differential diagnosis of patients with IBS-like symptoms [7]. Moreover, a primary and a secondary form of ISD exist; the first form depends on genetic conditions while the second is determined by duodenal villous atrophy and/or by inflammation [7].

Based on Roma IV criteria, IBS is classified according to the predominant stool pattern, recognized using the Bristol Stool Scale [2]. There are four different subtypes of IBS [8,9]:

- Constipation predominant IBS (IBS-C): more than 25% hard stools and less than 25% loose stools.
- Diarrhea predominant IBS (IBS-D): more than 25% loose stools and less than 25% hard stools. It appears to be the most common subtype, affecting approximately 40% of patients [10].
- Mixed bowel habits IBS (IBS-M): more than 25% loose stools and more than 25% hard stools.
- Unclassified IBS (IBS-U): less than 25% loose stools and less than 25% hard stools.

Moreover, symptoms, such as abdominal pain, have to be recurrent at least 1 day/week in the last 3 months (but with the onset at least 6 months earlier) associated with two or more of the following criteria [1,8,11]: (1) related to defecation; (2) associated with a change stool frequency; (3) associated with a change in the shape of the stool.

The aim of this review is to evaluate not only the clinical efficacy of the nutritional protocols usually suggested for IBS–D but also the effects of the diet on the intestinal microbiota modulation. First of all, it may be appropriate to briefly clarify the composition and functions of intestinal microbiota in healthy conditions. Since the literature on this topic is very heterogeneous, this review has mainly focused on studies concerning the adult population; moreover, the effects of prebiotics, probiotics or symbiotics supplementation and of fecal transplantation were excluded.

## 2. Human Intestinal Microbiota: A Brief Description of the Composition and Functions

In 2001, the Human Genome Project was completed [12,13,14]. Thus, thanks to the findings of the Human Microbiome Project (HMP) and the Metagenome of Human Intestinal Tract (MetaHIT), microbiologists have understood the existence of a profound relationship between human and human-associated microbial communities. Many symbiotic bacteria colonize human body, where the main colonization sites are: mouth, gut, vagina and skin. Of these four sites, the human gut microbiota is the most relevant. It is composed of a variety of bacteria, archaea, microeukaryotes and viruses and the gut one is constituted by ten trillion of different symbionts (50 bacterial phyla and about 100–1000 bacterial species). Collective microbiota genes formed the microbiome, which is 150 times larger than the human genome [15,16].

The composition of the human microbiota is influenced by the host genotype, the environment and the diet [17]. The intestinal microbial ecosystem balance is called eubiosis, while a system in which bacteria no longer coexist together in mutual harmony is described as dysbiosis [18]. An intestinal microbiota in an eubiotic state is characterized by the preponderance of potentially beneficial species, belonging mainly to the two bacterial phylum *Firmicutes* and *Bacteroidetes*, while potentially pathogenic species, such as the phyla *Proteobacteria* (*Enterobacteriaceae*), are present but in a very low percentage [19].

The intestinal microbiota must be considered a real organ, with well-defined functions [20]. The gut microbiota is in a mutualistic relationship with the host, harvesting additional energy and nutrients from the diet and protecting the host from pathogens. It produces a wide range of metabolites and chemicals, which influence host functions [21]. As stated by Arumugam et al., in healthy adults, three basic enterotypes have been described in the gut microbiota. Each enterotype has a distinctive cluster of bacteria and functions: bacteria belonging to the enterotype cluster 1 (high percentage of genera Bacteroides) derive energy mainly from carbohydrates using principally glycolysis and pentose phosphate pathways while bacteria belonging to the cluster of enterotypes 2 and 3 (high percentage of *Prevotella* and *Ruminococcus*, respectively) are able to degrade mucin glycoproteins of the gut mucosal layer [22]. The microbiota has several roles such as (1) barrier against hexogen microbes, (2) structural and metabolic functions [23] and (3) development and activation of immune system [24,25].

Research on the human gut microbiota has predominantly been based on the analysis of fecal samples. Actually, the mucosal microbial composition is very different from the fecal one, which represents the luminal microbiota [26,27]. Stools are considered a surrogate for the luminal content of the GI tract but their composition does not necessarily reflect the microorganisms adhering to the mucosa. Several studies have reported that the fecal-associated microbiota differs from mucosal-associated microbiota [26,28]. In particular, *Bacteroidetes* are more abundant in the mucosal-associated microbiota instead of *Firmicutes, Actinobacteria, Bacilli* and *Proteobacteria*, which dominate the fecal-associated microbiota [29].

The gastrointestinal tract is anatomically and functionally divided into the esophagus, stomach, small and large intestine [30]. Although the stomach was thought to be sterile, healthy human stomach is normally inhabited by five major phyla—*Firmicutes, Bacteroidetes, Actinobacteria, Fusobacteria* and *Proteobacteria—*with a predominance of the following bacterial genera: *Prevotella, Streptococcus, Veillonella, Rothia* and *Haemophilus* [31]. Looking at the small intestine, the predominant phyla in the duodenum are *Firmicutes* and *Actinobacteria*. The jejunum supports the growth of Gram-positive aerobes and facultative anaerobes (103-7 CFU/mL) including *Lactobacilli, Enterococci* and *Streptococci* [32]. In the ileum, the bacterial density reaches up to 109 CFU/mL with a predominance of aerobic species; on the contrary, the distal part of the ileum is populated by anaerobes and Gram-negative organisms, more similar to the colon environment [30]. The large intestine (LI), composed of the ascending, transverse and descending colon and the cecum, is inhabited by a number of anaerobes that exceeds 100–1000 aerobes [28].

*Firmicutes* and *Bacteroidetes* mainly dominate the LI and the bacterial density reaches 1012 CFU/mL [26]. The *Firmicutes* phylum is composed of 301 phylotypes and more than 200 different genera such as *Lactobacillus, Bacillus, Clostridium, Enterococcus* and *Ruminococcus* and most (95%) of its sequences belong to the *Clostridia* class [27,33,34].

The *Firmicutes/Bacteroidetes* ratio is considered a predictive marker of health or disease and can be modified at different stages of life and in pathological conditions [35]. Focusing on the predominant bacterial genera, in the LI lumen there are: *Bacteroides*, *Bifidobacterium, Streptococcus, Enterobacteriaceae, Enterococcus, Clostridium, Lactobacillus* and *Ruminococcus*, while *Clostridum, Lactobacillus, Enterococcus* and *Akkermansia* are associated with the mucosa [36]. The phylum *Proteobacteria* represents a small percentage of GI bacteria in healthy individuals [36]. Few differences were observed between the ascending and descending colon in terms of microbiome composition [37]. A mucus-stratified layer covers the colic mucosa, where organisms that can use mucin such as *Akkermansia, Ruminococcus* and *Bacteroides* are present [38]. A limited number of pathogens like *Campylobacter jejuni*, *Salmonella enterica, Vibrio cholera, E. coli, Bacteroides thetaiotaomicron* and *Bacteroides fragilis* may be present in LI with less abundance (0.1%) [39,40].

## 3. Fecal and Mucosal Microbiota in IBS, with a Focus on IBS-D

Bacteria, fungi and protozoa are all implicated in the development of IBS. Colonization of the non-resident microbiota and an overgrowth of the resident microbiota in the small intestine (a relatively sterile portion of the gut) could be associated with IBS symptoms. Regarding protozoa, *Blastocystis hominis* seems to be associated with the IBS–D subtype [41]. Most of the existing literature on the intestinal microbiota composition in IBS-D patients has highlighted a significant modification of *Actinobacteria, Firmicutes, Bacteroidetes* and *Proteobacteria.*

In IBS-D patients, the data regarding *Actinobacteria* and *Bifidobacterium* are conflicting: several studies have demonstrated a significant reduction in both fecal [42,43,44,45] and mucosal [46] samples. In particular, Zhong et al. showed a significant reduction of *Bifidobacterium* [47], in particular of *Bifidobacterium faecis* [48], in the mucosal microbiota of these patients. In contrast to this evidence, two studies showed a greater abundance of *Actinobacteria* in IBS-D fecal microbiota [29,46].

Regarding the *Firmicutes* phylum, two studies showed greater abundance in IBS-D patients compared to healthy controls [42,46], especially for *Bacilli* [29], in particular *Lactobacillales* (order) [49] and *Lactobacillaceae* (familia), both in fecal and in mucosal microbiota [50]. To *Lactobacillaceae* belong *Pediococci* [27], *Lactobacilli* [27,42,43,46,51], *Streptococci* [49] that are increased in IBS-D patients and *Lactococci* that are reduced in fecal samples of these patients [52]. *Firmicutes* also include the class of *Clostridia* that increases in IBS-D patients [29], but within this class, *Subdoligranulum* [49] and *Anaerovorax* [49] in the mucosal microbiota, *Faecalibacterium* [27,49]—especially *Faecalibacterium prausnitzii* [29,44,53]—both in fecal and in mucosal microbiota, and *Clostridium leptum* [45] only in the fecal one—are reduced in IBS-D patients compared to healthy controls. Conflicting data are reported on the *Lachnospiraceae* family belonging to the *Clostridiales* as Krogius-Kurikka et al. reported that there is a significant increase in IBS-D [42] while Ringel et al. showed a reduction of this family in both mucosal and fecal microbiota [47]. Other studies have also reported an increased abundance of *Dorea* spp. [29,51] and *Coprococcus* [29] and a reduction of *Anaerostipes* [52].

It has also been shown that both the composition of the fecal and the mucosal microbiota in IBS patients are poor in *Bacteroidetes* compared to healthy controls [29]. Looking at the genus of *Bacteroides*, Ringel et al. showed a significant reduction of *Bacteroides* [49] meanwhile two studies showed an increase of this class, especially *Bacteroides vulgatus* and *Bacteorides fragilis,* both in mucosal and in fecal, and *Bacteroides thetaiotaomicron* only in the fecal one [43,49]. The stool fecal [29,42,44,49] and mucosal samples [29,46,48] of IBS patients also showed a significant reduction of *Prevotella, Alistipes* and *Tennerella* compared to healthy controls.

Interestingly, Lo Presti et al. showed an increased abundance of *Parabacteroides distesonis*, a species that belonged to *Parabacteroides,* both in mucosal and in fecal microbiota [52].

Regarding the phylum of *Proteobacteria*, two studies have shown an increased abundance in IBS-D patients compared to healthy controls [29,46]. Carroll et al. and Maccaferri et al. demonstrated an increased concentration of *Enterobacteriaceae,* especially *Escherichia coli*, both in the fecal [27,50] and in the mucosal microbiota [46,47] and an increased abundance of *Fusobacterium* in fecal samples [27].

All these evidences confirm the complexity in identifying a specific pathological profile of the intestinal microbiota in order to better orient the therapeutic approach.

## 4. Correlation between Intestinal Microbiota and IBS Symptoms

Several metabolic functions of the intestinal bacteria could modulate the onset and/or the worsening of the GI symptoms in IBS.

*Bifidobacteria* and *Lactobacilli*, for example, are known to exert a beneficial effect since they produce SCFAs, via carbohydrates fermentation [54], which acidify the luminal environment, inhibiting adherence of invasive bacteria [55,56]. Actually, *Bifidobacteria* and *Lactobacilli* are commonly used as probiotics thus helping to prevent colonic inflammation and to maintain a stable intestinal microecology [57]. Both *Bifidobacteria* and *Lactobacilli* could also produce broad-spectrum antibacterial substances such as lipophilic molecules, bacteriocins and hydrogen peroxide, as demonstrated in animal models, that play an important role in the intestinal tract competing for nutrients, producing acid metabolites and reducing the pH level thus inhibiting or destroying pathogens as *E. Coli* and *Salmonella* [58]. For their properties, *Bifidobacteria* seem to be effective in the treatment of loose stools while Lactobacilli could be targeted against high defecation frequency [47].

There is also a correlation between loose stools and diarrhea and higher levels of bile acids (BAs) in the colon [59].

BAs, excreted into the small intestine via the biliary duct, play a pivotal role in the absorption of dietary fats [60]. Interestingly among microbiota’s functions metabolization of BAs was found [61]. Unfortunately, a state of dysbiosis alters the physiological functions of BAs and this alteration seems to be implicated in IBS-D pathophysiology [62]. Dior et al. demonstrated that fecal primary bile acids, including chenodeoxycholic acid which has laxative properties, were increased in IBS-D patients compared to healthy controls and bile acid deconjugation activity was decreased in IBS-D feces [61]. IBS-D patients are characterized by a BAs malabsorption and by their accumulation into the colon [62], that causes diarrhea by several mechanisms: stimulating sodium and water secretion, inducing mucus secretion, increasing colon motility, stimulating defecation and/or damaging the mucosa with decreased intestinal permeability [59].

Intestinal micro-inflammation seems to play a crucial role in the pathophysiology of IBS [63], being responsible of a subsequent neuro-modulation that could generate GI symptoms as abdominal pain.

*Clostridiales* (*Firmicutes*), *Bacteroides* and *Escherichia Coli* (*Proteobacteria*) are opportunistic bacteria that, in specific pathogenic conditions, could produce a large amount of toxins provoking the activation of intestinal inflammation responsible for the onset of abdominal pain and diarrhea [47]. These bacteria, when colonizing the submucosa, dissolve glycoproteins and rapidly affect the production of colonic mucus causing an alteration of the intestinal barrier function, an increase of the intestinal permeability responsible of a significant mucosal translocation of pathogens [47]. By colonizing the submucosa, they can also induce a systemic immune reaction and a local inflammatory response, especially in the colon [47]. Moreover, it is well known that the Lipopolysaccharide (LPS), a component of the cell wall of *E. coli* (Proteobacteria), through the activation of TLR4 receptors on the surface of the immunocytes, can promote the release of pro-inflammatory factors, inducing an important inflammatory reaction and enhancing the intestinal mucosal permeability [47].

Therefore, many pathogens in the *Clostridium* genus appear to be associated with symptoms such as diarrhea, abdominal distension and abdominal pain [64]. Producing several toxins, toxigenic *Bacteroides fragilis* affects cellular pathways, interferes with cell proliferation, damages DNA and promotes the release of inflammatory factor from epithelial cells, and these mechanisms seem to be involved in the genesis of inflammation and cancer [65]. In contrast, non-toxigenic *Bacteroides fragilis* inhibits the intestinal inflammation and maintains the balanced immune system by producing Polysaccharide A [66].

Other bacteria have an anti-inflammatory action, for example *Faecalibacterium prausnitzii*, which is one of the butyrate-producing bacteria that contribute in maintaining the mucosal integrity and reduces the adhesion and colonization of pathogens in the intestinal tract [47,67]. Thus, it seems to be promising in ameliorating IBS symptoms [53].

Many studies have shown that *Prevotella* was associated with immunological imbalance in both localized and systemic diseases, probably through the promotion of mucosal Th17 immune responses [68,69,70]. Actually, less microbial richness and methane production, as well as a reduced prevalence of *Methanobacteriales* and *Prevotella* enterotype, were observed in subjects with severe IBS and, interestingly, the prevalence of *Prevotella enterotype* decreased with the increasing symptoms severity, in parallel with the increased prevalence of Bacteroides enterotype [46].

Another significant alteration in IBS-D patients is represented by increased gas production that has been associated with abdominal pain and flatulence [51]. It has been demonstrated that the *Lachnospiraceae* taxa could contribute to the general symptoms of IBS [71]; in particular, it seems that *Dorea* genus was associated with increased gas production and increased intestinal permeability, which is thought to contribute to IBS pathophysiology [29,51,72].

Patients with visible abdominal distension and symptoms like abdominal bloating/discomfort have also an increased abundance of *Clostridium coccoides* [43] and *Bacteroides thetaiotaomicron* [43]. In fact, colonization by *Clostridium* determines an excessive gas production, which is associated with IBS [73].

Most *Bacteroides* species, including *Bacteroides thetaiotaomicron,* have a multi-protein system associated with the cell-envelope, referred to as the “starch utilization system” that allows *Bacteroides* species to degrade carbohydrates and proteins [74]. More *Bacteroides* species may be associated with an excess organic acid production through fermentation [75], which could be associated with IBS symptoms.

IBS-D symptoms could be also associated with an alteration of the metabolism of Tryptophan (Trp), which is a biosynthetic precursor of a large number of microbial and host metabolites [76]. Trp metabolism in the gut includes the direct transformation of Trp by intestinal microorganisms into several molecules, such as indole and its derivates, many of which are ligands for aryl hydrocarbon receptor (AhR) [77]. Moreover, it is also responsible of the serotonin (5-hydroxytryptamine [5-HT]) production pathway in enterochromaffin cells via Trp hydroxylase 1 (TpH1) [78]. AhR signaling is considered a key component of the immune response at barrier sites and is crucial for intestinal homeostasis acting on epithelial renewal and barrier integrity [79]. Only few commensal species can produce AhR ligands, such as *Lactobacillus* spp. [80]. 5-HT, as stated before, is an important gastrointestinal signaling molecule that conveys signals from the gut to intrinsic or extrinsic neurons and influences intestinal peristalsis and motility, secretion, vasodilatation and the absorption of nutrients [81]. Gut microbiota is a major actor in the intestinal 5-HT production but the mechanisms by which the gut microbiota modulates 5-HT production are not fully understood [78]. There might be connections with physiopathology of IBS-D and impaired Trp metabolism [81] as 5-HT colon contents are increased in IBS-D [82].

Among Trp metabolites, there are indoleacetic acid (IAA) and indolepropionic acid (IPA) that affect the intestinal permeability, the host immunity [80] and the dysmetabolism of tryptophan indole derivatives (IAA and IPA), contributing to the pathogenesis in IBS-D [83].

Table 1 summarizes the microbiota composition in healthy subjects and symptoms in IBS-D subjects. 

## 5. Nutritional Protocols Used for IBS-D

Diet seems to play a pivotal role in IBS patients and 84% of them reported that their symptoms were linked to the foods they ingested [84,85,86], particularly fermentable carbohydrates and fats [86]. Moreover, some patients try to modify autonomously their usual diet by excluding some categories of foods that cause symptoms, thus leading to nutritional and energetic deficiencies [87,88]. Different foods could be responsible of several effects such as osmotic, chemical, mechanical and neuroendocrine effects but also of fermentation and of an alteration in the intraluminal pH and microbiome [89].

For this reason, a nutritional education in the choice of the foods most suitable for them is often a valid support in the patient care [90]. Researchers have sought an optimal dietary strategy for managing IBS but there is little evidence for IBS-D.

### 5.1. Traditional Dietary Advices

The first-line approach for IBS patients is based on changing eating habits and lifestyle. Dietary modifications are based on guidelines provided by the National Institute for Health and Care Excellence (NICE) and the British Dietetic Association (BDA) [91,92]. These guidelines emphasize the importance of regular meals, fiber and fluid intake as well as a proper intake of fatty and spicy foods and a limited consumption of alcohol and caffein [93].

To date, an “ideal diet” for IBS subjects has not yet been identified, so clinical practitioners usually suggest general advice such as [88,90,94]:

- To have regular meals and moderate portion sizes;
- Not to skip meals;
- To drink almost 2 L of liquids a day preferably water or caffeine-free drinks;
- To drink less than three cups of coffee or tea per day;
- To reduce alcohol or fizzy drinks;
- To moderate the fiber intake as it may be linked to the onset of symptoms. Among the fibers, they should reduce the insoluble one (e.g., bran or bran-based cereals, wholemeal flour or derivatives). Soluble fibers, on the contrary, should be recommended such as oat or Psyllium (Ispaghula);
- To reduce the consumption of “resistant starch” that is usually in pre-cooked or pre-packaged foods;
- To limit the consumption of fresh fruit to no more than three daily portions of 80 g each;
- To avoid sorbitol, an artificial sweetener found in chewing gums, in sugar-free drinks and in some products, especially for diabetic subjects.

Especially for IBS-D patients it is important to control the assumption of fructose and sorbitol, coffee (independently of caffeine content) and alcohol [88] and they should avoid aloe vera (for its laxative properties), acupuncture and reflexology [90].

Among traditional dietary advices, in Italy practitioners usually suggest to follow Mediterranean diet indications. It provides high consumption of fruit, vegetables, whole grains, legumes, nuts and seeds, fish, eggs, white meats and dairy products and a low consumption of red meats and processed meats. It seems to be associated with low incidence of cardio-vascular diseases and low levels of plasma cholesterol. Specifically, about dairy products—such as cheese and yogurt—fish, shellfish and white meats, it is recommended to consume them moderately, while red and processed meats are rarely consumed, and two eggs are recommended once a week [95].

### 5.2. Low-FODMAP Diet

The low-FODMAP (Fermentable Oligosaccharides, Disaccharides, Monosaccharides And Polyols) diet provides the reduction of consumption of short chain carbohydrates that are not absorbed in the small intestine and that are fermented in the colon [96]. FODMAPs are in wheat, milk and dairy products, in some kinds of fruits and vegetables, in legumes, in artificial sweeteners and in packaged foods [96]. This diet has been evaluated for a long time because it helps in reducing symptoms in IBS patients, especially those with IBS-D [96].

Eswaran et al. conducted the first randomized controlled trial in US comparing 4 weeks low-FODMAP diet vs. a modified NICE (mNICE) diet [97]. They used questionnaires to evaluate symptoms in 84 patients following the two different diets. IBS-D patients (Rome III criteria) were allocated to mNICE diet or Low-FODMAP diet. The first one provided to eat small, frequent meals, no trigger foods and to avoid excessive consumption of alcohol and caffeine without excluding FODMAPs that were markedly reduced in the low-FODMAP diet. Both diets determined a significant relief of symptoms in 40–50% of patients but the low-FODMAP diet determined a major reduction of symptoms like abdominal pain and stool consistency compared to mNICE diet. During this investigation, changes in quality of life, anxiety, depression, work productivity, activity impairment, sleep quality and fatigue were also evaluated: the low-FODMAP diet showed a significant improvement of all these items except for work productivity that had no changes with both diets [98]. Moreover, the FODMAP diet turned out to be threefold to fourfold less effective in IBS-D patients that present sucrase—isomaltase gene variation (both one and two gene copies) [6].

Zahedi et al. conducted a randomized controlled trial comparing a low-FODMAP diet versus traditional nutritional indications for 6 weeks. Both diets exerted an important relief of the GI symptoms and bowel habits with a significant effectiveness of low-FODMAP diet. Regarding QoL there was no significant difference between the two nutritional protocols from the baseline to the end of the study [5]. Goyal et al. recently published the first prospective and randomized trial on 101 IBS-D patients that were initially randomized in two arms (strict low-FODMAP diet with no more than 0.5 g of FODMAP per serving and a diet according to traditional dietary advices) for 4 weeks. Following this first treatment, from week 4 to week 16, a modified low-FODMAP diet and traditional dietary advices diet were administered. Ninety-nine patients concluded the phase I (4 weeks) with more than 50% of compliance, so they entered phase II (weeks 4–16). The results of this study showed that low-FODMAP diets, both strict and modified, improved symptoms and quality of life (reducing also medications). Moreover, the compliance was high and, at the end, even if not all subjects were able to gradually reintroduce FODMAPs, 64.1% succeeded in it maybe because they were greatly motivated to improve their quality of life [99].

Orlando et al. conducted a long–term study on low-FODMAP diet in 20 IBS-D patients. This study evaluated symptoms, with the Symptom Severity Scale (IBS-SSS), some inflammatory markers (C-reactive protein, cyclooxygenase—2 and prostaglandin E2) and the erythrocyte—membrane fatty acids (FA) composition by a lipidomic examination following a personalized low-FODMAP diet for 90 days. The results of this investigation showed a significant reduction of symptoms severity, an improvement of bowel movements and a significant concentration of prostaglandin E2. Moreover, they also observed a reduction in n-6 PUFA/n-3 PUFA ratio and a minor level of main fatty acids in the red-blood cell membranes [100].

### 5.3. Gluten-Free Diet (GFD)

Celiac disease prevalence can be higher in IBS-D than other IBS subtypes, but IBS symptoms can exist also in treated subjects and they reduce after a gluten-free diet [101]. Celiac disease/gluten sensitivity and IBS-D are distinct entities with some overlapping symptoms [102]. Recent studies have showed that IBS patients have a worsening of symptoms after gluten/wheat ingestion so the consumption of these substances and gluten sensitivity have be considered involved in the etiology of IBS [103,104,105,106]; thus, a gluten-free diet is usually linked to a reduction of GI symptoms in IBS [92]. However, Guidelines 2020 recommended to verify if IBS-D patients are affected by celiac disease, performing the serological tests before giving a diet [9]. It is not clear if it is gluten that determines symptoms or other wheat components. Thus, nowadays the term wheat sensitivity (WS) seems to be preferred and the HLA—DQ2/8 genotype is not useful to diagnose WS. Barmeyer et al. showed that 34% of 35 non-celiac subjects with wheat sensitivity (21 HLA—DQ2/8 negative and 14 HLA—DQ2/8 positive) had a relief of symptoms after a GFD that did not contain wheat [107].

Aziz et al. confirmed this correlation in his prospective study conducted on IBS –D patients. They have evaluated the effects of 6 weeks GFD on 41 non-celiac patients. Twenty subjects were HLA—DQ2/8 positive and 21 HLA—DQ2/8 negative. The results have shown that GI symptoms changed significantly in 71% of the population independently from their HLA-DQ group. Against their expectations, they have observed a more significant reduction in abdominal pain in HLA—DQ2/8 negative than positive ones while items linked to psychological conditions changed significantly in HLA—DQ2/8 positive subjects. Some patients continued the GFD even after the end of the study for 18 months reporting an ongoing symptoms remission [108].

In another interesting RCT, 45 non-celiac subjects were randomized to GFD or GCD (gluten-containing diet) creating subgroups according to HLA-DQ2/8 positive or negative genotype. They have showed that GFD had a significant decrease in stool frequency especially in the HLA—DQ2/8 positive group but there were no changes in stool form, in gastric empty and in colonic transit in both groups. Finally, they have also observed that the diet containing gluten determines an increased permeability in the small bowel mucosa (reducing *zonula occludens 1*) and in the rectosigmoid mucosa (reducing *zonula occludens 1, claudin 1* and *occludin*), as assessed by mannitol recovery. Effects on the intestinal barrier, evaluated by a minor production of mucosal tight junction proteins, are more visible in HLA—DQ2/8 positive than HLA—DQ2/8 negative subjects [109]. Wu et al. also observed a pathological alteration of tight junction with a significant variation of the phosphorylation of myosin II regulatory light chain (MLC), *claudin—15* and *claudin—2* following a gluten-free diet that is linked with a higher intestinal permeability. A positive correlation between an increase of MLC phosphorylation and an increased mannitol excretion was also observed [110].

### 5.4. Very Low Carbohydrate Diet (VLCD)

Austin et al. conducted the first and unique study that evaluated the effects of a Very low Carbohydrate Diet (VLCD) with no more than 20 g of carbohydrate/day in 13 subjects with overweight or obesity and IBS-D according to Rome II criteria. Participants followed 2 weeks of standard diet and then 4 weeks of VLC diet. The outcomes regarded an adequate relief of symptoms in almost 2 weeks of the 4 weeks of VLC diet, a reduction in bowel movements and abdominal pain and changes in stool consistency [111]. Moreover, the study has also evaluated the Quality of life and the sickness profile using specific questionnaires. All participants have showed a significant reduction of symptoms in almost 2 weeks of VLCD and 77% of them declared an improvement of symptoms in all 4 weeks, especially in the last week. At the end of the study, also the QoL and sickness profile improved after VLCD [111].

### 5.5. Lactose-Free Diet

Lactose is a disaccharide and it is broken down by lactase enzyme in galactose and glucose. The enzyme is located on the apical portion of the small intestine brush border. The lactase activity physiologically decreases with advancing age [112]. In lactose intolerant (LI) people, lactose is not digested and absorbed in the small intestine, thus it comes intact into the colon where it is fermented by the intestinal microbiota, producing SCFAs and other gases that determine abdominal distension and symptoms like diarrhea, bloating and abdominal pain [113].

IBS patients, especially the IBS-D subgroup, usually exclude lactose from their diet as, following the consumption of food containing lactose (e.g., dairy products), they have a significant worsening of symptoms [113]. Thus, many IBS-D patients prefer to consume lactose-free products [114] not knowing that self-reported milk intolerance is not a good diagnostic predictor of LI [115]. The prevalence of lactose intolerance seems to be significantly higher in IBS-D patients than in healthy individuals although it is not always associated to malabsorption [115,116,117].

It has been demonstrated that IBS-D patients with lactose intolerance are characterized by an increase of mast cells in the intestinal mucosa and an increase of serum TNFa and rectal sensitivity after lactose ingestion, compared to patients with only lactose malabsorption or healthy controls [116].

Yang et al. compared lactose adsorption in healthy volunteers and IBS-D patients and found that the risk of lactose intolerance was related to ingested lactose dose that determines a worsening of symptoms as intestinal gas production [118]. Generally people with lactase deficiency can tolerate up to 20 g of lactose but lactose intolerant subjects must also control the amount they tolerate and the symptoms they present [119]. A double-blind cross-over study compared the response of confirmed lactose malabsorbers to lactase and placebo demonstrating no association between symptoms severity and lactase treatment, and suggesting that IBS symptoms were independent of lactose maldigestion [120].

Despite this little evidence, a recent review has showed that there is no evidence to suggest an objective link between IBS and lactose malabsorption [121].

### 5.6. Fructose-Free Diet

A fructose-free diet is usually suggested in subject with fructose malabsorption [122] or with positive fructose breath test [123]. Choi et al. conducted a study on 80 IBS patients of which 26 with fructose breath test positive: all fructose intolerant subjects reported diarrhea/loose stools that was not present in fructose tolerant IBS patients. Only 14/26 fructose intolerant patients had a relief of symptoms (abdominal pain, belching, bloating, fullness, indigestion and diarrhea) with a fructose-restricted diet for approximatively 1 year [123].

## 6. Effects of Different Nutritional Approaches on the Intestinal Microbiota

The microbiota and its modifications following different nutritional approaches are extensively studied but clinical evidence is less clear and generally lacking. It is therefore known that different nutritional protocols can modify the microbiota causing benefits or symptoms [124]. To evaluate the effects of different kind of diets on the microbiota we have considered both studies on healthy subjects and on IBS patients focusing on IBS-D whenever possible. Unfortunately, to date, few studies have been performed evaluating microbiota modifications in the IBS-D.

### 6.1. Traditional Dietary Advices

CAFFEINE: Coffee may directly affect the host’s gastrointestinal physiology by increasing intestinal motility and reducing intestinal transit time [125,126]. Coffee contains some non-nutritional compounds such as phenolic compounds, fibers, minerals and caffeine [127]. From these compounds, caffeine, (poly)phenols and fibers can reach and exert some of their effects in the large intestine, being fermented by the gut microbiota [128]. González et al. conducted a study to evaluate the effects of caffeine on the microbiota including 147 healthy participants which were categorized into three groups: non-coffee-consumers (0–3 mL/day), moderate consumers (3–45 mL/day) and high-coffee consumers (45–500 mL/day). Higher levels of *Bacteroides-Prevotella-Porphyromonas* has been observed in high consumers [129].

SWEETENERS: Sweeteners alter the gut microbiota; however, the effects on the microbiota composition have not been fully elucidated. Within Non-Sugar Sweeteners (NSSs), only saccharin and sucralose shift the populations of gut microbiota. Ingestion of saccharin showed alterations in metabolic pathways related to glucose tolerance and dysbiosis. However, further studies are needed to clarify these preliminary observations. Several polyols, including iso-malt and maltitol, increase *Bifidobacteria* numbers in healthy subjects, and these polyols can have prebiotic actions. On the other hand, different human clinical trials have shown that lactitol decreases the populations of *Bacteroides, Clostridium, coliforms* and *Eubacterium* [130].

RESISTANT STARCH: Resistant starch (RS), moreover, is not digested and resist to the passage through the stomach and small intestine, reaching the colon intact where it can be fermented by specialized microorganisms such as *Rumiococcus bromii* and *Bifidobacterium adolescentis* [131].

SPICES: Spicy foods, especially those containing capsaicin (CAP), have recently attracted considerable attention from the perspective of their positive action on the intestinal flora, eliminating the enteric pathogens, and promoting the growth of beneficial bacteria [132,133]. A short-term, high-dose CAP-enriched diet (10 mg/day, for two weeks) increases the abundance of *Faecalibacterium* in healthy humans [134]. This suggests that CAP is a determinant factor for *Faecalibacterium*’s presence in the intestinal flora [135].

FIBER: Fibers are carbohydrates that arise from the plant cell wall and resist gastric acidity, enzymatic hydrolysis and absorption in the upper gastro-intestinal tract [136]. Fibers are trophic to the bacteria that form the intestinal microbiota and they act as prebiotics. In this way, the bacteria are able to produce metabolites useful for human health [137]. Accessible microbiota carbohydrates (MACs) are carbohydrates that resist digestion and are made available to the gut microbiota as metabolizable prebiotics into SCFAs [138].

Generally, they are divided into soluble and insoluble although there are some “fibrous foods”, such as psyllium or oats, which contain a certain amount of both soluble and insoluble fibers. Fibers should be classified according to their fermentability, viscosity and gel-forming ability, for example:

- insoluble and not very fermentable fibers (i.e., whole grains);
- soluble, non-viscous and readily fermentable fibers (i.e., inulin);
- soluble fibers capable of forming gels and non-fermentable (i.e., psyllium).

Fast fermenting fibers can lead to gas formation, such as methane and hydrogen, while poorly fermentable and the non-fermentable ones could lead to lower gas production [136,139]. Soluble fibers are often recommended as they have multiple properties such as helping in case of diarrhea because they make the stool more compact while insoluble fibers are not recommended in this subtype [140]. Unfortunately, consuming fiber also determines an increase of the osmotic effect and, consequently, an increase of the colic contents volume with distension of the abdominal walls, responsible of symptoms generation [141,142]. This problem appears to be mainly related to a consumption of insoluble rather than soluble fibers [140,143,144]. In fact, Moayyedi et al. showed a significant improvement in symptoms of IBS patients following a nutritional intervention that involved a soluble fiber supplement [145], while the effect of insoluble fiber has not been investigated. A diet that involves the consumption of fibers from fruit and legumes is associated with a great diversity of the microbiota and a predominance of *Prevotella* over *Bacteroides* [146].

### 6.2. Low-FODMAP Diet

The low-FODMAP (LFD) diet is the better treatment option for symptom relief in IBS, but this diet leads to a reduction of health-associated bacteria (i.e., *Bifidobacteria*) and causes a dysbiotic microbiome composition [147]. Sloan et al. reported that a low-FODMAP diet has an impact on intestinal microbiota composition, with conflicting data [148]. Restricting FODMAPs intake reduces the relative abundance of Actinobacteria, predominantly *Bifidobacteria* and *Clostridia* (particularly *Lachnospiraceae*), many of which are butyrate producers [148,149]. On the contrary, Dieterich et al. showed that *Lachnospiraceae* increased with a low-FODMAP diet [150]. Valeur et al. explored whether gut microbial composition may identify symptoms response to a 4-week low-FODMAP diet in patients with IBS: among 61 patients enrolled, 32 were classified as responders (high adherence to low-FODMAP diet) and 29 as non-responders (low adherence to low-FODMAP diet). The responders had significantly high levels of *Bacteroides fragilis, Acinetobacter, Ruminiclostridium, Streptococcus* and *Eubacterium* and lower levels of *Clostridia/Negativicutes/Bacilli, Actinomycetales, Anaerotruncus, Clostridiales* and *Shigella/Escherichia* [151]. Hustoft et al. investigated the effects of low-FODMAP diet on symptoms and gut microbiota composition in IBS-D and IBS-M adults: 20 patients with diarrhea-predominant or mixed IBS were instructed to follow a low-FODMAP diet (LFD) throughout a 9-week study period. After LFD were found a reduction of *Clostridium, Faecalibacterium prausnitzii, Bifidobacterium, Megasphaera, Pediococcus* and *Actinobacteria*, and a significant increase abundance of *Dorea* [152]. Studies with a better distinction among IBS subtypes are needed.

### 6.3. Gluten-Free Diet

The composition of the gut microbiota is susceptible to the influence of the diet, and especially to the quality and quantity of ingested carbohydrates. Several studies have observed that, following a GFD over 1 month the gut microbiota of healthy subjects showed a decrease of *Bifidobacterium* spp. (especially *B. longum*), *Clostridium lituseburense* and *Faecalibacterium prausnitzii* and a significant increase of *Enterobacteriaceae* and *Escherichia coli* [153,154,155]. The reduction of *B. longum*, after gluten-free diet could be explained by the fact that more than 8% of this genus is involved in carbohydrate and polysaccharide metabolism [156]. Bonder et al. showed that after GFD, the strongest gut microbiota variations occur in the family of *Veillonellaceae*. They have also demonstrated a significant decrease of *Ruminococcus bromii* and *Roseburia feces* in GFD while *Victivallaceae, Clostridiaceae,* and *Coriobacteriaceae* families were increased [157]. Moreover, several investigations have also shown an increase of pathogenic species such as *Enterococcus, Staphylococcus, Salmonella, Shigella* and *Klebsiella*, influencing the microbial profile having an important effect on the long-term homeostasis of the intestinal mucosa of healthy subjects [153,155,157,158].

A randomized, controlled, cross-over trial involving 60 middle-aged healthy Danish adults with two 8-week interventions comparing a low-gluten diet (2 g gluten per day) and a high-gluten diet (12 g gluten per day) shows that a low-gluten diet induces changes in their intestinal microbiome [159]. The low gluten group was characterized by a lower abundance of four species of *Bifidobacterium* and *Dorea* (especially *Dorea longicatena*), one species of *Blautia wexlerae*, two species of the *Lachnospiraceae* family, and two butyrate-producing bacteria—*Anaerostipes hadrus* and *Eubacterium halii—*in comparison with the high-gluten diet [159]. At the same time, an unclassified species of unknown taxonomic origin, an unclassified species of *Clostridiales* and an unclassified species of *Lachnospiraceae* increased during the low-gluten diet intervention compared with the high-gluten diet intervention [159]. Studies on IBS-D population are needed.

### 6.4. Low-Carbohydrate Diet

A randomized controlled trial explored the effect of carbohydrate restriction on the intestinal microbiota composition of IBS patients, compared to an IBS population which did not receive a dietary intervention, demonstrating a significant reduction in concentration and proportion of luminal *Bifidobacteria* [160]. On the contrary, levels of total bacteria and the amount of *Bacteroides, Prevotella, Eubacterium rectale, Clostridium coccoides, Faecalibacterium prausnitzii, Lactobacillus* and *Enterococcus* remained unchanged after this nutritional intervention [160]. Therefore, many authors have described a decreased microbial diversity induced by the Ketogenic diet (KD) [161]. This is the result of reduced carbohydrate intake, which led to a decrease in polysaccharides content, determining a decrease of bacteria that produce energy from polysaccharides. Moreover, KD appears to reduce blood glucose levels and body weight, and to increase blood ketone levels [161]. These effects were correlated with the increase of SCFA producers bacteria such as *Akkermansia muciniphila* and *Lactobacillus* and with the reduction of pro-inflammatory taxa such as *Desulfovibrio* and *Turicibacter* [158,161]. Studies on IBS-D subjects are needed.

### 6.5. Lactose-Free Diet and Fructose-Free Diet

The effects of a lactose/fructose-free diet on healthy, IBS or IBS-D adults microbiota have not been evaluated in the current literature.

### 6.6. Mediterranean Diet

Gutiérrez-Díaz et al. explored the association between the adherence to a Mediterranean dietary pattern and its components and fecal microbiota composition in a cohort of healthy adults [162]. High adherence to MD was associated with a significant presence of phylum *Bacteroidetes*, family *Prevotellaceae* and genus *Prevotella*, which is able to hydrolyze xylan and cellulose [163] and inversely related with the phylum *Firmicutes*, the family *Lachnospiraceae* and the genus *Ruminococcus* (*Ruminococcaceae* family). Moreover, some specific MD components were linked to the increase of speficic gut taxa, such as cereals with the genera *Bifidobacterium* and *Faecalibacterium*, olive oil with *Tenericutes* and *Dorea*, red wine with *Faecalibacterium*, vegetables with *Rikenollaceae, Dorea, Alistipes* and *Ruminococcus* and legumes with *Coprococcus* [162]. Studies on IBS-D population are needed.

## 7. Other Nutritional Protocols

### 7.1. Vegan and Vegetarian Diet

The vegetarian diet is very rich in fiber, and low in animal food protein and saturated fats; it is based on foodstuffs of plant origin, such as cereals, legumes, root, crops, oilseeds, fruit, vegetables, nuts and mushrooms. The vegetarian diet is also rich in phytoestrogens, antioxidants and n-3 fatty acids [164]. On the other hand, a vegan diet is different from a vegetarian diet because it does not include animal products (i.e., eggs or dairy products), but at the same time is similar to vegetarian diet because has a high intake of fruits and vegetable and has a low intake of both sodium and saturated fat [165]. Vegetarians’ intestinal microbiota is rich in *Bifidobacterium* and *Lactobacillus* and poor in pathogenic *Bacteroides fragilis* and *Clostridium* perfringens. This composition determines an increased production of short-chain fatty acids (SCFA) levels [166]. The vegan microbiota profile appears to be unique in several characteristics, including a reduced abundance of pathobionts and a greater abundance of protective species [167]. Matijašić et al. showed that both vegetarian and vegan subjects were associated with a higher ratio of the *Bacteroides*-*Prevotella* group and also had a lower ratio of *Clostridium* cluster XIVa when compared to omnivores [168]. Studies on IBS-D population are needed.

### 7.2. Western Diet

The western pattern diet is a dietary habit chosen by many people in the developed countries, and increasingly in the developing countries [169]. This dietary pattern is characterized by high intake of red meat, simple sugars, high-saturated fat foods and refined grains [170]. It also typically contains high-fat dairy products, high-sugar drinks [171] and high intake of processed meat. Usually, the assumption of important vitamins and minerals, which are essential for good health, is not typically included in this diet [172]. In humans, a high intake of dietary fat (mainly saturated fatty acids) is associated with reduced microbiota richness and diversity [173]. A recent study showed that a high-fat diet in healthy adults is associated with increased levels of *Alistipes* and *Bacteroides* spp. and a decrease in *Faecalibacterium* spp. [174]. A long-term animal protein-rich diet is associated with an increase of *Bacteroides* [175]; on the other hand, a short-term animal protein-rich diet increases the levels of bile-tolerant bacteria spp., such as *Alistipes, Bilophila* and *Bacteroides*, while decreasing the abundance of *Roseburia* spp., *Eubacterium rectale* and *Ruminococcus bromii* [176]. Studies on IBS-D population are needed.

In Table 2, the effects of different nutritional protocols on intestinal microbiota in healthy or IBS patients are summarized.

## 8. Summary

IBS-D is a chronic functional gastrointestinal disorder in which the pathogenesis is multifactorial and still unclear. Several factors have been involved in the genesis of IBS, such as structural abnormalities in the jejunal epithelial barrier gene mutations of sucrase—isomaltase [6,7], and a different production of proteins involved in digestion and metabolism, especially after fat and protein foods as well as fermentable carbohydrates [6,7,177].

Moreover, an alteration in the composition of microbiota could also be among causes of IBS-D [2]. The present review evaluated both fecal and mucosal microbiota composition of this IBS subgroup, which are characterized surely by a reduction of *Bifidobacteria* [42,43,44,45,47], in particular, *Bifidobacterium faecis* in fecal one [48], *Faecalibacterium* [27,49]—especially *Faecalibacterium prausnitzii* [29,44,53]—and *Clostridium leptum* [45] only in the fecal composition, compared to healthy subjects.

Each phylum has a specific function, so we could hypothesize that its reduction or increase could determine an improvement or a worsening of IBS-D symptoms.

For example, *Dorea* (that belonged to *Firmicutes*), is a carbohydrate utilizing specie that produces gas and this production is associated with abdominal pain and flatulence; moreover, it has been demonstrated that this species is related to an increased intestinal permeability typical of IBS-D [29,51,72]. Some of these studies have reported an increased abundance of *Dorea* spp. in IBS-D patients compared to healthy subjects [29,51], hypothesizing a possible correlation between IBS-D symptoms and an increased abundance of *Dorea*.

Not only gas is produced from carbohydrate fermentation but also SCFAs [178]; in fact, these metabolites are the result of oligosaccharide, galactooligosaccharides (GOS), fructooligosaccharides (FOS) and polysaccharide inulin fermentation made by *Bifidobacteria* and *Lactobacilli* [54]. SCFAs—such as acetate, propionate and butyrate—acidify the luminal environment inhibiting the adherence of invasive bacteria i.e., *Clostridi* (*E. coli* and *Salmonella*) [55,56,58]. Among SCFAs, butyrate is biologically the most relevant and its receptor (GPR109A) is expressed in the lumen-facing apical membrane of colonic and intestinal epithelial cells [179]. IBS is characterized by a disruption of gut integrity and butyrate is a key promoter of colonic health and integrity [180], contributing to the microbial homeostasis [181,182].

Interesting evidence suggests that *Bifidobacteria* could be targeted in the treatment of loose stools while *Lactobacilli* could be effective in restoring the defecation frequency [47].

Moreover, among *Firmicutes*, there is interesting evidence regarding *Faecalibacterium prausnitzii* that provides the main energy source for intestinal epithelial cells by producing butyrate, which supports the integrity of the intestinal mucosa [47,67] and reduces adhesion and colonization of pathogens in the intestinal tract [47]. This species has also immunomodulatory and anti-inflammatory properties [67] and seems to have promising effects in ameliorating IBS symptoms [53]. Several investigations have shown lower levels of *Faecalibacterium prausnitzii* in IBS-D patients compared to healthy controls, both in fecal and in mucosal microbiota [29,44,53], suggesting a possible relation with a worsening of IBS-D symptoms.

Exclusion diets should not be suggested in IBS-D patients except for the low-FODMAP diet [183], which is considered the pivotal dietary treatment option for symptom relief in IBS [148,149]. The beneficial action of the low-FODMAP diet is probably caused by the fact that it is low in MAC [96], thus reducing the production of gas often responsible for abdominal pain and bloating. Nevertheless, it has not been suggested in the long-term because it can cause dysbiosis [147] by reducing the relative abundance of *Actinobacteria*, predominantly *Bifidobacteria* and *Clostridia*, many of which are butyrate producers [148,149]. A reduction of *Bifidobacteria* seems to be associated with abdominal pain in healthy individuals [184] and in IBS subjects [185]. To date, the low-FODMAP diet should not be considered a valid long-term nutritional protocol for IBS-D. Further RCT studies are needed to evaluate an intervention over a longer period and its effects on the microbiota [5].

The role of VLCD remains to be elucidated with more RCTs despite the positive effects shown in the study of Austin et al. [111]. The long-term effects on both symptoms and changes in the gut microbiota also need to be evaluated [111].

A vegetarian diet is associated with bacterial diversity [33] while a vegan one is associated with a reduced abundance of pathobionts and a greater abundance of protective species [167]. Vegetarians’ intestinal microbiota is rich in Bifidobacterium and Lactobacillus and poor in pathogenic *Bacteroides fragilis* and *Clostridium perfringens*. This composition determines an increased production of short-chain fatty acids (SCFA) levels [166].

The vegan diet, on the one hand, increases the microbiota richness and, on the other hand, worsens IBS symptoms, probably for the increase of *C. Coccoides* that is associated to abdominal distension and bloating/discomfort [43].

Lactose or fructose exclusion is not recommended in IBS-D patients with no malabsorption or intolerance of these sugars. However, hydrogen breath test should be conducted in IBS-D patients to exclude a lactose or fructose malabsorption that could generate symptoms.

## 9. Conclusions

In conclusion, the relationship of IBS symptoms and food is complicated; moreover, although different nutritional approaches are popular for IBS, there is little scientific evidence, so more indications come from clinical practice [186].

There is interesting evidence that changes in nutritional habits could lead to a relief of gastrointestinal symptoms. At the same time, there are no data confirming a specific nutritional protocol that could promote eubiosys and a reduction of IBS-D symptoms with a good patient compliance [33].

Although the low-FODMAP diet is the better treatment option for IBS-D, because it can have a favorable impact on symptoms (especially abdominal pain, bloating and diarrhea [148]), this nutritional protocol leads to a reduction of health-associated bacteria (i.e., Bifidobacteria) and reduces the relative abundance of many butyrate producers [147,148,149]. The gluten-free diet also reduces IBS-D symptoms but determines a dysbiotic microbiome composition [153,154,155]. Hence, it is very difficult to choose a nutritional protocol that at the same time is able to reduce IBS-D symptoms and return to eubiosis; further studies are needed to obtain this goal.

In the current literature there are few clinical trials that evaluate the correlation among nutrition, microbiota and symptoms in IBS subjects, but most of these studies do not specify the IBS subtype. Observing a single IBS subtype could be important to define the best nutritional approach that could lead both to an improvement of specific symptoms and the microbiota composition.

On the basis of the few evidences present, the relationship between dietary habits and the composition of the intestinal microbiota must be better explored in order to identify, in these patients, specific nutritional strategies capable of contributing to the relief of symptoms by modulating the intestinal microbiota.

## Figures and Tables

**Table 1:** Microbiota composition in Healthy Subjects (HS) and symptoms in IBS-D subjects.

Phylum and Functions	Genera	HS	IBS-D Subjects	Symptoms
***Firmicutes*** Degrade mucin glycoproteins of the gut mucosal layer [22] Produces butyrate that contribute to maintainthe mucosal integrity and reduces the adhesion and colonization of pathogens in the intestinal tract (*Faecalibacterium)* [47,67]	*Ruminococcus* [22] *Lactobacillus* [27] *Clostridia* [33] *Dorea* spp. *Coprococcus* [29], *Anaerovorax, Subdoligranum,* *Faecalibacterium, Anaerostipes* [52]	✓	↑ *Clostridia, Dorea* spp., *Coprococcus* [29,50,51] ↓ *Lactobacillus, Anaerovorax, Subdoligranum, Faecalibacterium (F. prausnitzii)* [27,33,49]	Diarrhea, abdominal distension, abdominal pain and excessive gas production (*Clostridia*) [64,73] Increases gas production and intestinal permeability and contributes to IBS pathophysiology (*Dorea* spp.) [29,51,72]
***Bacteroidetes*** Derives energy primarily from carbohydrates using principally glycolysis and pentose phosphate pathways [22]	*Prevotella* [22] *Bacteroides* *Tennerella* *Alistipes* *Parabacteroides*	✓	↑ *Bacteroides thetaiotaomicron, B.vulgatus, B. fragilis, Parabacteroides (P. distesonis)* [52], ↓ *Prevotella, Alistipes, Tennerella* [29,46,48,49]	Increased symptoms severity associated to a reduction of *Prevotella* [46] and are associated with excess organic acid production (*Bacteroides)* [75]
***Actinobacteria*** Beneficial effect producing SCFAs [54] and broad-spectrum antibacterial substances [58]	*Bifidobacterium* [36]	✓	↓ [29,46]	Reduced antinflammatory effects for reduced SCFAs production [54]
***Proteobacteria*** Pathogens [36]	*E. coli* [36] *Salmonella enterica* [36] *Vibrio cholerae* [36]	✗	↑ *Enterobacteriaceae (E. Coli)* [27,50]	Abdominal pain and diarrhea [47]

✓ the phylum is normally present in a healthy subject microbiota composition; ✗ the phylum should not be present in a healthy microbiota composition; ↑ species increased ↓ species decreased.

**Table 2:** Effects of different nutritional protocols on the intestinal microbiota in healthy subjects or IBS patients.

Type of Nutritional Protocol	Author		Type of Study	Population	Duration of the Study	Changes in Microbiota Composition
**Traditional nutritional advices**	González et al., 2020 [129]	Caffeine	Intervention study	Healthy adults *n* = 147	1-year	↑ *Bacteroides-Prevotella-Porphyromonas* [in high-consumer of coffee]
	Kang et al., 2016 [134]	Capsaicin	Intervention study	Healthy adults *n* = 12	6-weeks	↑ *Faecalibacterium*
**Low-FODMAP diet**	Sloan et al., 2018 [148]		Single-center, Parallel group, double-blind RCT	Healthy adults *n* = 37	14-days	↓ *Bifidobacteria and Clostridia (especially Lachnospiraceae)*
	McIntosh et al., 2016 [149]		RCT, single-blind study	IBS Adults *n* = 37	3-weeks	↓ *Bifidobacteria* ↑ *Actinobacteria*
	Dieterich et al., 2018 [150]		Case-control	19 NCGS Adults and 10 healthy controls *n* = 29	4-weeks	↑ *Lachnospiraceae*
	Valeur et al., 2018 [151]		Controlled-feeding, transversal study	IBS Adults *n* = 61	4-weeks	↓ *Clostridia/Negativicutes/Bacilli, Actinomycetales, Anaerotruncus,, Clostridiales* and *Shigella/Escherichia* ↑ *Bacteroides fragilis, Acinetobacter, Ruminiclostridium, Streptococcus* and *Eubacterium*
	Hustoft et al., 2016 [152]		Intervention study	IBS-D and IBS-M adults *n* = 20	9-weeks	↓ *Clostridium*, *Faecalibacterium prausnitzii*, *Bifidobacterium*, *Megasphaera*, *Pediococcus* and *Actinobacteria* ↑ *Dorea*
**Gluten-free diet**	De Palma et al., 2009 [153]		Controlled-feeding, transversal study	Healthy adults *n* = 10	4-weeks	↓ *Bifidobacterium longum, Clostridium lituseburense* and *Faecalibacterium prausnitzii* ↑ *Enterobacteriaceae, E. coli, Staphylococcus, Salmonella, Shigella* and *Klebsiella*
	Sanz, 2010 [155]		Controlled-feeding, transversal study	Healthy adults *n* = 10	4-weeks	↑ *Enterococcus, Staphylococcus, Salmonella, Shigella* and *Klebsiella*
	Bonder et al., 2016 [157]		Controlled-feeding, transversal study	Healthy adults *n* = 21	4-weeks	↓ *Veillonellaceae, Ruminococcus bromii* and *Roseburia faecis* ↑ *Victivallaceae, Clostridiaceae Coriobacteriaceae, Enterococcus, Staphylococcus, Salmonella, Shigella* and *Klebsiella*
	Hansen et al., 2018 [159]		Randomized, controlled, cross-over trial	Healthy adults *n* = 21	>2 months	↓ *Bifidobacterium, Dorea, Blautia, Lachnospiraceae, Anaerostipes hadrus* and *Eubacterium halii*
**Low-Carbohydrate diet**	Staudacher et al., 2012 [160]		RCT	IBS Adults *n* = 35	4-weeks	↓ *Bifidobacterium*
**Mediterranean diet**	Gutiérrez-Díaz et al., 2016 [162]		Controlled-feeding, transversal study	Healthy adults *n* = 31	n.d.	↓ *Firmicutes, Lachnospiraceae* and *Ruminococcus* ↑ *Bacteroidetes, Prevotellaceae, Faecalibacterium, Tenericutes, Dorea, Rikenollaceae, Alistipes, Ruminococcus* and *Coprococcus*
**Vegetarian diet**	Matijašić et al., 2014 [168]		Controlled-feeding,	Healthy adults *n* = 60 (31 vegetarian and 29 omnivorous)	1-year	↓ *Clostridium cluster XIVa* ↑ *Bacteroides/Prevotella*
**Western diet**	Wu et al., 2011 [175]		Controlled-feeding	Healthy adults *n* = 10	10-days	↑ *Bacteroides*
	David et al., 2014 [176]		Controlled-feeding	Healthy adults *n* = 10	5-days	↓ *Roseburia* spp., *Eubacterium rectale and Ruminococcus bromii* ↑ *Alistipes, Bilophila* and *Bacteroides*

## Data Availability

Not applicable.
